# Use of Culturally Focused Theoretical Frameworks for Adapting Diabetes Prevention Programs: A Qualitative Review

**DOI:** 10.5888/pcd12.140421

**Published:** 2015-05-07

**Authors:** Vetta L. Sanders Thompson, Michelle Johnson-Jennings, Ana A. Baumann, Enola Proctor

**Affiliations:** Author Affiliations: Michelle Johnson-Jennings, University of Minnesota College of Pharmacy, Minneapolis, Minnesota; Ana A. Baumann, Enola Proctor, Washington University in St. Louis, Brown School, St. Louis, Missouri.

## Abstract

**Introduction:**

Diabetes disproportionately affects underserved racial/ethnic groups in the United States. Diabetes prevention interventions positively influence health; however, further evaluation is necessary to determine what role culture plays in effective programming. We report on the status of research that examines cultural adaptations of diabetes prevention programs.

**Methods:**

We conducted database searches in March and April 2014. We included studies that were conducted in the United States and that focused on diabetes prevention among African Americans, American Indians/Alaska Natives, Asian Americans/Pacific Islanders, and Latinos.

**Results:**

A total of 58 studies were identified for review; 29 were excluded from evaluation. Few adaptations referenced or followed recommendations for cultural adaptation nor did they justify the content modifications by providing a rationale or evidence. Cultural elements unique to racial/ethnic populations were not assessed.

**Conclusion:**

Future cultural adaptations should use recommended processes to ensure that culture’s role in diabetes prevention–related behavioral changes contributes to research.

## Introduction

Almost 29 million US adults have diabetes, and as many as 86 million have prediabetes (1). The high rate of diabetes among US minority populations is concerning because diabetes is a risk factor for cardiovascular disease, vision loss, end stage renal disease, disability, and mortality (2). From 2010 through 2012, African Americans (13.2%), American Indians/Alaska Natives (AI/ANs) (15.9%), Asian Americans and other Pacific Islanders (9.0%), and Latinos (12.8%) were more often diagnosed with diabetes than were non-Hispanic whites (7.6%) (1). Diabetes is preventable through lifestyle changes that may also assist in diabetes control.

The Institute of Medicine (IOM) examined the impact of social and cultural environments on health outcomes and recommends that research advance in this area (3). According to the IOM report, health behaviors and other social variables occur in a cultural context that must be understood to determine which cultural variables influence adoption of health recommendations. 

There is evidence that interventions (eg, for cancer care, mental health, health education) that emphasize integration of cultural knowledge (ie, ideas, rules of etiquette, and knowledge needed in social life) improve outcomes among adults (4–6). Emerging data suggest similar effects in diabetes interventions (7). Although data on cultural adaptations for youths are equivocal (5) and concerns have been raised about the impact and consequences of constituency involvement in assessments of cultural appropriateness for public health interventions (8), further evaluation is warranted to determine the key factors affecting outcomes.

Castro et al (9) suggest that the aim of cultural adaptations should be “to generate a culturally equivalent version of a model prevention program” when elements in the original intervention produce resistance to program activities or are in conflict with cultural attitudes. Castro et al (5) identified steps to guide decisions to culturally adapt evidence-based interventions, which involves justification of the effort. Justification for adaptation may be based on previous failure to engage members of priority populations or the presence of unique cultural risk factors and symptoms, or both. Once justified, an evidence-based intervention is selected and cultural adaptations of content and delivery are completed (5).

Frameworks for cultural adaptations have emerged in 2 forms. One form involves modification within content categories (10–12), with early discussions emphasizing “surface” and “deep structures” of modification (11). “Surface structure” modifications involve inclusion of photos, symbols, and recruitment and outreach strategies (11). Resnicow et al refer to “deep structure” as recognizing, reinforcing, and building on a group’s values and behaviors to provide context and meaning to important intervention components (11). The framework proposed by Kreuter et al further specifies surface and deep cultural elements (10). Culturally sensitive programming requires changes to peripheral, evidential, linguistic, constituent-involving, and sociocultural categories (10). Peripheral approaches focus on colors, fonts, photographs, or declarative titles. Linguistic strategies assure that all intervention materials are in the preferred language of the group (12). Evidential approaches make use of testimonials, narratives, stories, and statistics specific to the group and raise awareness of perceived vulnerability to the health issue (10). Constituent-involving strategies include hiring or training group members or from the community or extensively engaging the community (10), which takes advantage of members’ insider knowledge about the community’s health perceptions and may increase acceptability and relevance (13). Sociocultural approaches discuss disease in the context of social or cultural characteristics (eg, including traditional foods and physical activities) (10).

The second form of cultural adaptation frameworks defines the steps of the intervention adaptation process (5,9,14) and offers the opportunity for a systematic process. The PEN-3 model completes cultural adaptions in 2 phases that support community input on the appropriate adaptation elements. The first phase, assessment, involves information gathering to learn about the community and its perspective (the resources that promote [ie, nurturers] or inhibit [ie, barriers] behavioral change and the roles that friends and family play in behavioral change). Once this information is gathered, the community and researchers use assessment data to critique current strategies and collaboratively develop culturally appropriate interventions (14).

Barrera et al (6) reviewed the past decade’s literature to identify elements that are common to cultural adaptations of behavioral health interventions relevant for diabetes interventions. The authors report 5 stages of cultural adaptation that are a refinement of earlier recommendations: information gathering, preliminary design, preliminary testing, refinement, and final trials (3,6). The review suggests that interventions involving the inclusion of cultural elements in an adaptation are more effective than control or usual care conditions (6). The authors recommended that studies evaluate cultural adaptations completed in these stages. 

In this article, we examine the cultural adaptation of diabetes prevention programs and the extent to which the call for research advances in this area is being met. We also examine content and characteristics of cultural adaptations and the extent to which the recommended “how” and “what” of adaptation have been adopted. Recommendations for next steps are provided.

## Methods

The studies included in this review were compiled from a search of computerized databases conducted in March and April of 2014. The search performed was Academic Search Complete, and the following databases were selected: Academic Search Complete, CINAHL (*Cumulative Index to Nursing and Allied Health Literature)*, CINAHL Plus, Family and Society Studies Worldwide, Global Health, Global Health Archive, Medline, PsycINFO, and Social Work Abstracts. Research published from 2004 through 2014 was included to capture systematic research of cultural adaptations of diabetes prevention programs among ethnic minorities (3,6,10–12). Key words were used to search titles, abstracts, and subject headings in all databases. The Boolean search used key words, including “Diabetes Prevention Program” or “DPP” or “diabetes prevention” and “translation” or “translating” and “African American” or “African-American” or black or “American Indian” or “Native American” or “Latino” or “Latina” or “Hispanic” or “Asian” or “Asian American”; “Diabetes Prevention Program” or “DPP” or “diabetes prevention” and “translation” or “translating” and “sociocultural” or “cultural adaptation” or “sociocultural adaptation.” A supplemental search used the terms “PEN-3” and “deep culture” to identify additional articles.

Each study identified had to meet the following criteria for inclusion: 1) was a quantitative or qualitative research study completed in the United States; 2) had diabetes prevention as the primary focus, research question, or hypothesis of the study; 3) had diabetes education and interventions aimed at prevention activities, such as diet, exercise or physical activity, or health communication; and 4) included group-specific analyses on African Americans, AI/AN, Asian Americans/Pacific Islanders, or Latinos (although these priority populations did not have to be the only group studied). The reference lists of these articles were reviewed to identify other studies that met the inclusion criteria. Review articles, meta-analyses, dissertation abstracts, and articles in languages other than English were excluded from this evaluation. Journal articles reporting data from a single study were reported separately but evaluated as a single study.

Included studies were evaluated for 1) study population included; 2) diabetes prevention activity and program studied; 3) cultural adaptation process used; 4) formative research completed and analytic method (quantitative or qualitative) used; 5) cultural components and attributes (ie, peripheral, linguistic, evidential, sociocultural, constituent-involving) included to address values, attitudes, and behaviors; 6) inclusion of community strengths and resources in program or intervention; 7) channel or media selected or used in intervention; and 8) unique cultural elements assessed (eg, inclusion of spiritual factors, identity, rituals). Studies were coded by a graduate research assistant trained by the first author (V.L.S.). The first author then reviewed all studies and coding to resolve questions identified by the graduate research assistant or the author.

## Results

A total of 58 published manuscripts were initially identified; 29 were excluded from the evaluation. A total of 29 studies were included in the qualitative synthesis for this review (Figure).

**Figure:**
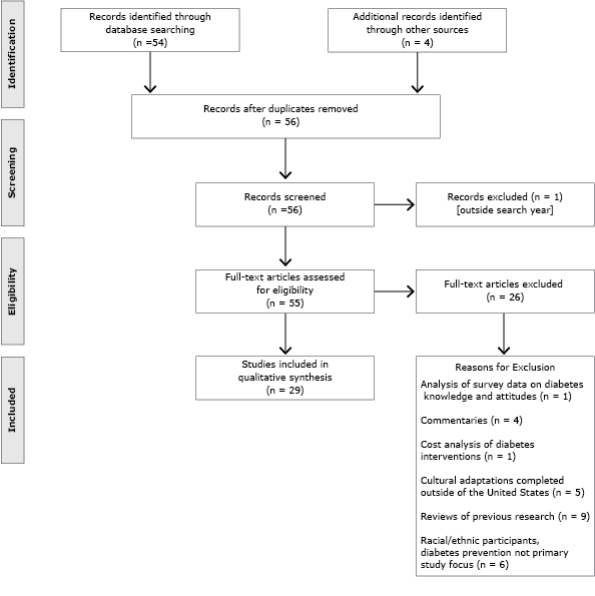
Number and reasons for article exclusion. Qualitative review of use of culturally focused theoretical frameworks for adaptations of diabetes prevention programs, United States, 2014.

Most studies addressed adaptation of diabetes prevention programs for Latinos (44.8%; Mexican, Puerto Rican, Dominican, and Caribbean) (15–27) and African Americans (31.0%) (28–36). Other adaptations were found for Asian Americans (2 studies: Korean, Filipino/Pacific Islanders) (37,38), AIs (4 studies: Northern Plains Indians, AI/ANs, urban southwest Indian) (39–42), and 1 study focused on a combined population (43) (Latinos/African Americans). One study focused on men (20), and 4 studies targeted women or involved mostly women (17,26,32,42) (Table 1).

**Table 1:** Summary of Diabetes Prevention Program Cultural Adaptations, by Race/Ethnicity, United States, 2014^a^

Characteristic	Latino (n = 13)	African American (n = 10)	American Indian/ Alaska Native (n = 4)	Asian American (n = 2)
**Demographic**				
Female only	2	1	1	0
Male only	1	0	0	0
Youth	1	2	3	0
**Program modified**				
Diabetes Prevention Program	8	6	4	2
Other	3	3	0	0
**Cultural adaptation**	13	9	4	2
**Adaptation uses theory^b^**				
Cultural	2	0	0	0
Other theory	7	3	1	2
**Study type**				
Formative only	4 (mean, 46.3 [range, 16–100])	1 (N = 25)	1 (N = 31)	1 (N = 127)
Pilot/feasibility	5 (mean, 31.4 [range, 12–91])	5 (mean, 32.8 [range, 8–62])	1 (N = 64)	1 (N = 48)
Trial	3 (mean, 175 [range, 69–312])	1 (N = 604)	1 (N = 2,553)	0
	Latino/African American, 1 (n = 183)			
**Level of adaptation^c^**				
Surface	4	3	2	2
Deep	13	6	3	2
**Outcome**				
Weight (eg, loss, BMI)	7	5	1	2
A1c, glucose, insulin sensitivity	2	2	0	1
Physical activity	4	3	1	1

Abbreviations: A1c, hemoglobin A1c; BMI, body mass index.

a Values are whole numbers unless otherwise indicated. Values in columns may not sum to total or may exceed total value for n, because studies could adapt to accommodate more than 1 attribute or could report more than 1 outcome.

b Theory-driven cultural adaptation process: C, cultural (PEN-3, Castro et al, 2010 [5]); OT, other theory/model (eg, community-based participatory research, social-cognitive theory, grounded theory).

c Level of adaptation adapted from Resnicow et al (11).

The Diabetes Prevention Program (DPP) was the dominant evidence-based program subject to adaptation (84.6%). Of the 7 non-DPP adaptations, 1 was based on a program (Group Lifestyle Balance Program) (19) that was an earlier adaptation of DPP. DPP was adapted for each of the racial/ethnic categories.

Despite the availability of guidelines for completing the cultural adaptation process (3,6,9,14) and identifying potential areas for content modification (8,9), few studies referenced these approaches to cultural adaptation (15,16). The studies using cultural adaptation used Barrera et al (6), with a reference to Resnicow et al (11) and Airhihenbuwa’s PEN-3 model (14). Eleven adaptations (17,20,22,23,26–28,36,37,39,43) used various other frameworks, with community-based participatory research (CBPR) most widely cited (24.1%) (Table 2).

**Table 2:** Detailed Summary of Diabetes Prevention Programs Evaluated for Cultural Adaptations, United States, 2014

Author	Population	Program Modified	Cultural Adaptation	Adaptation Process	Formative Studies	Content Category)^a^	Nurturer/ Barriers)^b^	Community Resources
Atkinson et al, 2009 (28)	African American	Church-based DPP	Yes	Grounded theory	Yes	E, S, C	N, B	Church
Boltri et al, 2011 (30)	African American	Group lifestyle DPP	Yes	—	No	S	N	Church
Boltri et al, 2008 (31)	African American	DPP	Yes	—	No	L, S	N	Church
Brown et al, 2010 (39)	Northern Plains, AI youth	DPP	Yes	CBPR	Yes	See below	N,B	Montana reservation
Brown et al, 2013 (40)	Northern Plains, AI youth	DPP	Yes	—	See Brown et al, 2010	P, L, E, S, C	N,B	Montana reservation
Burnet et al, 2011 (29)	African American (9-12 yrs)	Reach out	Yes	—	Yes	L, S	N, B	—
Chasan-Taber et al, 2014 (17)	Latina (pregnant)	Lifestyle intervention	Yes	Socio-cognitive/ TTM	Yes	L	B	—
Coleman et al, 2010 (18)	Latino Family	DPP	Yes	—	No	L, S	N, B	School
Cox et al, 2013 (32)	African American, women	DPP	Yes	—	No	C	—	—
Gutierrez et al, 2014 (43)	African American, Latino	DPP	Yes	CBPR	Yes	L, S	N, B	Church
Islam et al, 2013 (37)	Korean American	DPP	Yes	CBPR	Yes	P, E, L, S	N, B	—
Jiang et al, 2013 (41)	AI/AN youth	DPP	Yes	—	Yes	S	N, B	—
Kramer et al, 2013 (19)	Hispanic	GLB (DPP adaptation)	Yes	—	No	L, S	N	WIC
Mau et al, 2010 (38)	Filipino, Pacific Islander	DPP	Yes	CBPR	Yes	P,E, L, S,C	—	Gurdwara sites
Martinez et al, 2012 (20)	Male Mexican Immigrant	Formative	Yes	Socio-Ecological Model	Yes	L, S	N, B	—
Melancon et al, 2009 (16)	Mexican American and Mexican Native	Formative	Yes	PEN-3	Yes	S, C	N, B	—
Merriam et al, 2009 (see Ockene) (21)	Latino (Caribbean)	DPP	Yes	—	No	P, L, S	—	YWCA
Millard et al, 2011 (22)	Immigrant Hispanic	Diabetes Empowerment Education Program	Yes	CBPR, TTM, Socio-Ecological Model	No	L, S, C	N, B	—
Ockene, et al, 2012 (23)	Dominican/Puerto Rican Spanish speakers	DPP	Yes	Socio-cognitive theory	Yes	L, S	B	YWCA
Osuna et al, 2011 (15)	Latino/a	Mediterranean Lifestyle Program	Yes	Castro et al, 2010	Yes	P, L, S	N, B	—
Ramal et al, 2012 (24)	Latino/a, low-income	Formative	Yes	—	Yes	S	N, B	—
Ruggiero et al, 2007 (25)	Latino/a,	DPP	Yes	—	No	L, C	—	—
Ruggiero et al, 2011 (26)	Spanish speaking	DPP	Yes	CBPR	No	L, C	—	Community settings
Shaibi et al, 2012 (27)	Latino, adolescents	DPP	Yes	CBPR	No	S, C	N	YMCA
Sharma and Fleming, 2012 (33)	African American, youth	—	No	—	—	—	—	Community-based
Tang et al, 2014 (34)	African American	NDEP “Power to Prevent”	Yes	—	No	C	N	Church
Wells, 2011 (35)	African American	DPP	Yes	—	—	S	N,B	Church
Willging et al, 2006 (42)	American Indian, women, urban Southwest	DPP	Yes	—	Yes	P, S, C	N, B	—
Williams et al, 2013 (36)	African American	Fit Body and Soul	Yes	Socio-ecological	Yes	P, E, C	N	Church

Abbreviations: —, information unavailable or ambiguous; AI, American Indian; AN, Alaska Native; CBPR, community-based participatory research; DPP, Diabetes Prevention Program; NDEP, National Diabetes Education Program.

a Content categories: P, peripheral; L, linguistic; E, evidential; S, sociocultural; C, constituent involving.

b N, nurturers; B, barriers. Adapted from Airhihenbuwa (14).

Approximately 55.6% of studies conducted some form of information gathering or formative research in preparation for the cultural modification of an evidence-based program (15–17,23,24,28,31,36–43). Most studies collected qualitative data or used mixed methods. The primary data collection methods included focus groups for qualitative studies (n = 11) and surveys for quantitative studies (n = 4).

Four studies (25,32,34,36) focused only on surface adaptations of the intervention programs (10); an additional 7 combined surface and deep content modifications (15,21,26,37,38,40,42). Efforts included the use of community locations for meetings and organizations to assist in recruiting (21,26,34,36,38–40). Beyond churches (24.1%), the YMCA/YWCA (10.3%) was the most frequently identified community resource used in (primarily Latino) cultural adaptations. Five studies (17.2%) reported the use of racial/ethnic media for recruitment, dissemination of information, or education (21,28,31,37,38).

Of the studies completing adaptations of deep structure (n = 23), most (91.3%) used sociocultural adaptations (15,16,18–24,27–31,35–38,40,41,43), which included modifications of recipes, cooking and tasting demonstrations, recommendations for physical activity, leaders as role models and to deliver content, and the use of talking circles, storytelling, narratives, novellas, and soap opera video formats; this was followed by linguistic adaptations (61.5%), primarily for Spanish speakers (15,17–23,25,26,30,31,37,38,40,43). In all but 2 instances, language adaptations were combined with other changes. Modifications of evidential components occurred least often (19.2%) (28,36–38,40).

Approximately 52% of studies incorporated both nurturing elements of culture (promotes healthy behaviors) and cultural barriers (inhibits healthy behaviors) (15,16,18,20,22,24,28,31,35,37,39–43). Two studies (6.9%) focused solely on barriers (17,23), and 6 (20.7%) focused exclusively on nurturing elements (19,27,29,30,34,36). Nurturing elements focused on gaining support of elders and church leaders, prayer and spirituality, collectivism, and social support (14). Barriers focused on mistrust, privacy concerns, concerns about neighborhood safety and marginalization, and food traditions (14). No studies evaluated program components included as a part of a cultural modification.

Consistent with a recent review of DPP evaluations (44), 18 studies reported outcomes of cultural adaptation feasibility, pilot studies, and trials (13,18,19,22,23,25–27,29–33,37,38,40,41,43), with a primary outcome of weight loss. Seven studies from Latino communities reported weight loss (18,19,22,23,25–27) and improvement in hemoglobin A1c (23) and insulin sensitivity (27). The results of a family focused adaptation were mixed; weight loss and increased physical activity was reported among parents but not among youths (18). The church-based adaptation for Latinos and African Americans (43), 5 studies focused on African Americans (13,29–32), 2 on Asian Americans (37,38), and 1 AI/AN trial (41) reported similar weight loss findings. Two African American (29,30) and 1 Asian American study reported decreased blood glucose levels (37). Among African American studies, a family focused study (31) reported mixed findings, with changes among youths but not parents, and a youth intervention (33) resulted in changes in fat intake among boys but not girls. One AI study reporting a 3-month follow-up (40) failed to produce changes in body mass index.

## Discussion

This analysis suggests an increasing number of diabetes prevention cultural adaptations across racial/ethnic populations, reporting positive outcomes, primarily weight loss. The lack of comparisons to evidence-based interventions (no control or reliance on usual care controls) made it difficult to ascertain superior cultural adaptations. However, study data combined with the results of a recent diabetes treatment cultural adaptation (7) support the importance of continued research.

Few studies referenced recommendations for cultural adaptation processes or content. Given the recent emergence of some process recommendations, this is understandable (5,6); however, the PEN‑3 model (14) and content recommendations are older (10,11). Although the use of CBPR and various theoretical frameworks resulted in community input into cultural adaptations, a culturally focused approach may increase understanding of how specific cultural health beliefs vary across multiple populations and subpopulations (8) and aid in identification of key mechanisms for change (7).

Also of concern was the limited documentation of the rationale for modifications, as illustrated by Osuna et al (15) and the fact that only 52% of studies involved information gathering or a formative research phase to support the cultural modifications made to the original evidence-based diabetes prevention program. These data may have been reported as subpopulation research studies and may have been missed in our search, or authors omitted this information from study reports. However, a deliberative process should occur to avoid modifications informed by stereotypical or monolithic views of racial/ethnic communities. For example, it should not be assumed that all members of a Latino community speak Spanish as their primary language. Issues related to socioeconomics, religion, and sexual orientation should also be included.

That studies varied in their use of peripheral, linguistic, evidential, sociocultural and constituent-involving strategies is not surprising. As Osuna et al note (15), cultural adaptations should be restricted to issues and elements dictated by current research evidence and data emerging from the information-gathering phase. Although the types of modifications reported in studies seemed effective, the failure to measure participants’ responses to cultural elements is a lost opportunity to understand program acceptance and behavioral change.

Future diabetes prevention cultural adaptations should use recommended processes for cultural adaptation, including justification for the adaptation, the processes of formative research and information gathering and modification, modifications in response to data, reports of refinements based on preliminary studies, and the results of final testing (6). Detailed reporting of adaptations helps researchers develop information on common cultural program modifications and makes replication of the adapted intervention easier (45). To build evidence that diabetes prevention interventions that focus on integration of culture positively influence outcomes, studies should compare cultural adaptations to the original evidence-based intervention. Researchers should also evaluate unique cultural elements included in adaptations to determine their utility. Racial/ethnic groups are not monolithic and the cultural issues that affect their responses to health programs should be examined, with the process recommended by Castro et al (5) guiding efforts.
