# Erratum, Vol. 12, May 7 Release

**DOI:** 10.5888/pcd12.140421e

**Published:** 2015-05-28

A change to an author name in the byline of the article “Use of Culturally Focused Theoretical Frameworks for Adapting Diabetes Prevention Programs: A Qualitative Review” has been made. Dr Ana A. Baumann’s name was incorrectly spelled in the original release and has been corrected. The change was made to our website on May 12, 2015, and appears online at http://www.cdc.gov/pcd/issues/2015/14_0421.htm. We regret any confusion or inconvenience this error may have caused.

